# Pulmonary vein isolation durability with fluoroscopy or 3D mapping-guided radiofrequency balloon ablation: a mandated remap study

**DOI:** 10.3389/fcvm.2025.1525819

**Published:** 2025-04-10

**Authors:** Alexandre Almorad, Alvise Del Monte, Domenico Giovanni Della Rocca, Luigi Pannone, Charles Audiat, Roberto Scacciavillani, Lorenzo Marcon, Kazutaka Nakasone, Giampaolo Vetta, Ingrid Overeinder, Gezim Bala, Antonio Sorgente, Erwin Ströker, Juan Sieira, Sahar Mouram, Milad El Haddad, Amin Hossein, Ahmad Awada, Carlo de Asmundis, Gian-Battista Chierchia

**Affiliations:** ^1^ Heart Rhythm Management Centre, Universitair Ziekenhuis Brussel, Heart Rhythm Research Brussels, Postgraduate Program in Cardiac Electrophysiology and Pacing, Vrije Universiteit Brussel, European Reference Networks Guard-Heart, Brussels, Belgium; ^2^ Independent Researcher, Helsinki, Finland; ^3^ Independent Researcher, Brussels, Belgium; ^4^ Cardiology Department, Centre Hospitalier Saint Pierre, Brussels, Belgium

**Keywords:** paroxysmal arial fibrillation, pulmonary veins isolation durability, remapping, radiofrequency, 3D mapping

## Abstract

**Background:**

Effective balloon positionnking during pulmonary vein isolation (PVI) with a radiofrequency balloon (RFB) is crucial for optimal energy delivery, maximising lesion formation, and preventing gaps. Traditionally, fluoroscopy is used to guide pulmonary vein (PV) occlusion, however, this method exposes patients to radiation. Recently, RFBs equipped with 3D electroanatomical mapping (EAM) offer an alternative approach, potentially achieving the same results with reduced radiation exposure. Our main aim was to evaluate procedural characteristics, such as acute isolation and time-to-isolation (TTI), when the RFB is positioned based only on fluoroscopy feedback vs. fluoroscopy and a 3D-EAM. The secondary objective was to assess PVI durability through mandated remapping in asymptomatic patients from both groups.

**Methods:**

A total of 60 patients were enrolled and underwent either a fluoroscopy-guided (FLUO, 30 patients) or fluoroscopy + 3D-EAM (3D-MAP, 30 patients) ablation. In each group, 15 patients without any documented recurrence underwent protocol-mandated repeat 3D-EAM six months after the index ablation. Procedural outcomes, lesion metrics, and safety profiles were assessed and compared between groups.

**Results:**

At a median follow-up of 579 days, freedom from any atrial tachyarrhythmias (ATAs) was 89.7% in the FLUO group and 92.3% in the 3D-EAM group (*P* > 0.05). The latter was associated with significantly reduced fluoroscopy exposure (median 10.5 vs. 7.0 min, *P* < 0.005). Procedure time and efficacy metrics, including single-shot isolation rates and TTI, were comparable between groups. Durable PVI on a per PV basis was present in 54/60 (90%) vs. 57/60 (94%) of PVs in the FLUO and 3D-EAM groups, respectively (*P* = 0.9).

**Conclusion:**

Radiofrequency balloon led to a high rate of durable PVI whether its guided by fluoroscopy only or 3D mapping. The latter allowed avoiding dye comsuption and a reduction of fluoroscopic times.

## Introduction

Pulmonary vein isolation (PVI) is the cornerstone of atrial fibrillation (AF) ablation treatment (1). As the number of patients with AF requiring treatment continues to grow, it seems that the most efficient and reproducible approach is single-shot technologies (2). The standard procedure for a single-shot catheter utilizes cryoballoon ablation (CBA); however, a radiofrequency balloon (3) (RFB, Heliostar, Biosense Webster, CA, USA) has recently been introduced as an alternative option. Based on a previous study, compared to CBA, the RFB has shown similar safety, efficacy, and efficiency profile with shorter dwell and thermal delivery times (4).

Correct balloon positioning is critical to achieve effective energy delivery to the targeted pulmonary veins (PVs), thus maximising lesion formation and preventing gaps that can lead to arrhythmia recurrence (5). Optimal RFB positioning, including correct alignment with the PVs and electrode-tissue contact, is mainly assessed via fluoroscopy with real-time x-ray imaging, defining anatomical landmarks and observing balloon inflation and contrast dye injection (6). While fluoroscopy offers valuable visual feedback for RFB positioning, it has limitations when assessing the quality of tissue contact and the real-time effectiveness of the ablation. This is where advanced techniques, like 3D mapping-guided positioning, based on orientation, and tissue characteristics measurements, such as temperature and impedance, can provide additional accuracy and improve outcomes (7).

In addition, it is well-established that the primary mechanism for AF recurrence after conventional ablation procedures is electrical PV reconnection over time due to incomplete lesion transmurality and/or contiguity. The frequency of all durable PVIs per patient has been reported to range from ∼20% to 80% (8–10).

In this study, we compared the acute and long-term efficacy and safety of PVI of PV occlusion when guided by standard fluoroscopy vs. 3D mapping. In addition, we assessed the durability of electrical PVI using protocol-mandated invasive remapping procedures in patients without documented arrhythmia recurrence.

## Methods

### Study population

This was a prospective, single-centre study. Between May 2022 and June 2023, 60 consecutive patients with paroxysmal AF who were scheduled for PVI using the RFB (Heliostar, Biosense Webster, Inc., Irvine, CA, USA) were enrolled. The study adhered to the ethical principles outlined in the Declaration of Helsinki (2013 revised version) and was approved by the local ethics committee of the Universiteit Ziekenhuis Brussel (NCT06333327).

### Ablation procedure

Patients were treated under general anaesthesia and uninterrupted anticoagulation therapy. A circular multi-electrode oesophagal temperature monitoring probe (CIRCA) was positioned to ensure complete coverage of the oesophagal path.

In all patients, a diagnostic decapolar catheter was introduced and positioned inside the coronary sinus to monitor atrial activity and allow superior vena cava pacing during radiofrequency (RF) delivery in the right veins.

A single transseptal access was performed using a fixed sheath under transesophageal echography guidance and fluoroscopy guidance. Immediately after gaining access to the left atrium (LA), a bolus of heparin was administered to reach and maintain an activated clotting time (ACT) of 300–350 s throughout the procedure. After exchanging the sheath for a dedicated deflectable one (14F, Guidestar, Oscor), the RFB and circular catheter (LassoStar, Biosense Webster, CA, USA) were introduced into the LA. An electroanatomical map was created using the circular catheter.

### Fluoroscopy-guided vs. 3D mapping-guided balloon positioning

Sixty consecutive patients undergoing RFB-based PVI were assigned to each group. The first 30 patients in the FLUO group where the balloon was positioned using standard fluoroscopy. Specifically, the proper wedging of the balloon at the junction between the LA and PV was confirmed by the fluoroscopic confirmation that the contrast medium injected into the PV through the inner lumen of the RFB did not leak back into the LA (Figure 2, right panel) (3). During the positioning phase of the RFB at the PV ostias, the operator used solely a fluoroscopic approach and was blinded to the mapping system and all its parameters displayed (e.g., impedance and temperature).

The second half of patients (*n* = 30) underwent ablation using the 3D system (3D-EAM group). In this groups, as described previously (6), the RFB was carefully positioned at the PV ostias using fluoroscopy for balloon alignment with the PV and a mapping system visualisation to assess the following baseline parameters (Figure 2):

Electrode impedance (*Z*) 90–120 Ω with a variability ≤20 Ω across electrodes pre-ablation and electrode temperature (T) ≤31 °C with a variability ≤3°C between electrodes (4, 6). In this group, no contrast medium was used to assess occlusion.

Before starting RF delivery, operators were asked to identify and select the three electrodes facing the posterior wall. The power setting was 15 Watts and the target electrode temperature was 55°C. The same energy was simultaneously delivered to all electrodes, with a duration of 20 s for the posterior and 60 s for the non-posterior electrodes. The irrigation flow rate was 35 ml/min during RF energy delivery (5 ml/min when RF was off).

During ablation, PV potentials were monitored using a circular diagnostic catheter to evaluate real time-to-isolation (TTI). As defined previously (4), single-shot isolation was defined as a TTI of <12 s. In cases of longer times, an extra application, segmental or circumferential, was performed.

Acute isolation was defined as confirmed PVI validated with a multipolar catheter at the end of the procedure, and waiting time/adenosine proof was left at the operator's discretion.

The skin-to-skin time was defined as the time from the first puncture to the withdrawal of the last catheter. Dwell time was defined as the time the RFB spent in the LA. The oesophagal temperature monitoring strategy was described in a previous paper (11).

### Post-procedural management and follow-up

All patients underwent continuous telemetry monitoring for at least 24 h after the procedure and were discharged after overnight observation if no complications arose. Oral anticoagulation was initiated the same evening after ablation and continued for at least two months; thereafter, it was prolonged according to the patient's thromboembolic risk profile. Antiarrhythmic drugs (AAD) were discontinued at the latest one month after ablation.

Any major periprocedural complications were collected [e.g., death, atrio-oesophageal fistula, stroke/transient ischemic attack (TIA), pericardial effusion/tamponade with/without surgical treatment, myocardial infarction, pulmonary veins stenosis, and persistent phrenic palsy] occurring within seven days post-procedure (except for atrio-oesophageal fistula). Minor complications were also reported, including vascular access complications requiring treatment, pericarditis, and transient phrenic palsy.

The clinical follow-up strategy included at least three in-person outpatient visits at 3, 6, and 12 months post-ablation. Each visit included a clinical examination and 12-lead electrocardiogram (ECG). Furthermore, one seven-day Holter at 6 months and another 24-h Holter were recorded during the first 12 months post-procedure. Regular telephone consultations were conducted between scheduled visits.

### Repeat electrophysiology study

All patients underwent a seven-day Holter monitorisation at six months. In the case of undocumented arrhythmia, 15 patients from each group underwent protocol-mandated repeat electroanatomical mapping to evaluate the durability and level of the PVI. After femoral access and transseptal puncture, a high-density anatomical map was acquired with a voltage and activation map during distal coronary sinus pacing with the Carto 3 mapping system using a multipolar catheter (PentaRay, Biosense Webster, CA, USA). In the case of PV reconnection, the exact site(s) of PV-LA conduction were marked and then re-ablated with a 3.5 mm contact force RF catheter. Isolation was then reassessed with a new electroanatomical map.

### Study endpoints

The primary endpoint was to compare procedural outcomes between a 3D mapping strategy vs. a fluroscopy only strategy for RFB positioning during PVI. Efficacy outcomes included single-shot isolation, time-to-isolation (TTI), skin-to-skin time, dwell time, fluoroscopy time, and absence of any atrial tachyarrhythmias (ATas) >30 s during follow-up.

The secondary endpoint was to compare PVI durability between the two strategies via a protocol-mandated remapping evaluation.

The safety endpoints included adverse events occurring within 30 days of the ablation procedure, which included cardiac tamponade, diaphragmatic paralysis, stroke, death, heart block, myocardial infarction, and vascular access complications.

### Analysis and statistics

The results are presented as absolute values with percentages, medians, and interquartile ranges (IQR). Normally and non-normally distributed continuous variables are compared using the Student's *t*-test and Mann–Whitney *U* test, respectively, whereas categorical variables were compared using the *χ*^2^ test. The Kaplan–Meier estimator, the product limit estimator, was used to estimate and plot survival functions; time-to-event analysis was performed using the log-rank test (Mantel-Cox test). A two-sided *α* of less than.05 (*P* < .05) was considered statistically significant. All statistical analyses were performed using SPSS (Statistical Package for the Social Sciences) version 27.0 software (IBM SPSS Statistics) and GraphPad Prism version 10.2.2 (GraphPad Software, Boston, Massachusetts, USA).

## Results

### Clinical characteristics

Between May 2022 and June 2023, 60 patients were assigned to undergo PVI with the RFB using the FLUO protocol (30 patients) using the 3D-EAM protocol (30 patients). The two groups were comparable in terms of baseline characteristics (Table 1).

**Table 1:** Baseline demographic and clinical characteristics.

Characteristics	FLUO	3D-EAM
Number of patients, *n*	30	30
Age, years (range)	65.0 (59.0–71.0)	67.0 (57.0–75.0)
BMI, kg/m^2^ (range)	26.8 (24.1–32.8)	28.1 (24.3–33.8)
Male, *n* (%)	19.0 (63.3)	16.0 (53.3)
Type of atrial fibrillation, *n* (%)		
Paroxysmal	27.0 (90.0)	26.0 (86.7)
Persistent	3.0 (10.0)	4.0 (13.3)
Left ventricular ejection fraction %, (range)	55.0 (55.0–60.0)	55.0 (55.0–58.8)
CHA2DS2-VASc score (range)	1.0 (0.5–3.5)	3.0 (1.0–4.0)
Hypertension, *n* (%)	19.0 (63.3)	19.0 (63.3)
Diabetes Mellitus, *n* (%)	2.0 (6.7)	7.0 (23.3)
Coronary artery disease, *n* (%)	5.0 (16.7)	6.0 (20.0)
Congestive heart failure, *n* (%)	1.0 (3.3)	3.0 (10.0)
Stroke/transient ischaemic attack, *n* (%)	1.0 (3.3)	4.0 (13.3)
Chronic Kidney Disease, *n* (%)	2.0 (6.7)	3.0 (10.0)

### Procedural details, lesion metrics, and effectiveness

Procedure and dwell times, including post-ablation 3D mapping, were similar, with a median of 55.0 (44.5–65.0) vs. 62.5 (45.0–75.0) min, and 17.0 (16.0–25.0) vs. 21.5 (16.0–33.0) min in the FLUO and 3D-EAM groups, respectively. However, 3D-EAM patients showed significantly shorter fluoroscopy time [10.50 (8.5–16.0) vs. 7.00 (5.5–14.5) min (*P* < 0.01)].

There was no difference in single-shot isolation between the two groups. In addition, the TTI was comparable for all veins. The complete procedural characteristics of both groups are summarised in Table 2.

**Table 2:** Procedural outcomes between the two groups.

Procedural Outcomes	FLUO	3D-EAM	*P*-value
Skin-to-skin procedure time, m (range)	55.00 (44.50–65.00)	62.50 (45.00–75.00)	0.6
Left common PV, *n* (%)	3.0 (10.0)	2.0 (6.7)	0.9
Median overall TTI, s (range)	9.0 (8.0–11.0)	9.5 (8.0–11.0)	—
Median TTI by vein, s (range)			
LSPV	10.00 (8.75–12.00)	11.00 (8.00–12.00)	0.9
LIPV	9.00 (7.50–10.50)	9.00 (7.75–9.25)	0.7
RIPV	9.00 (7.75–10.00)	9.00 (8.00–10.00)	0.9
RSPV	8.00 (7.00–10.00)	8.00 (7.00–10.00)	0.8
Single-shot isolation by vein, *n* (%)			
LSPV	25.0 (83.3)	28.0 (93.3)	0.3
LIPV	28.0 (93.3)	27.0 (90.0)	0.3
RIPV	26.0 (86.7)	30.0 (100.0)	0.4
RSPV	28.0 (93.3)	28.0 (93.3)	0.8
Fluoroscopy time, min (range)	10.50 (8.50–16.00)	7.00 (5.50–14.50)	0.003
LA dwell time, min (range)	17.00 (16.00–25.00)	21.50 (16.00–33.00)	0.2
AAD at discharge, *n* (%)	2.0 (6.7)	4.0 (13.3)	0.5
AAD after one year follow-up, *n* (%)	3.0 (10.0)	5.0 (16.7)	0.7
ATAs after one year follow-up, *n* (%)	3.0 (10.0)	2.0 (6.7)	0.6

AAD, antiarrhythmic drugs; ATAs, atrial tachyarrhythmias; LA, left atrium; LIPV, left inferior pulmonary vein; LSPV, left superior pulmonary vein; RIPV, right inferior pulmonary vein; RSPV, right superior pulmonary vein; TTI, time-to-isolation.

No major periprocedural complications occurred in either group. Phrenic nerve capture was transiently lost in one patient from the FLUO group and none from the 3D-EAM group. The patient had recovered phrenic nerve function at the one-month follow-up visit.

### Follow-up outcomes

**Figure 1:**
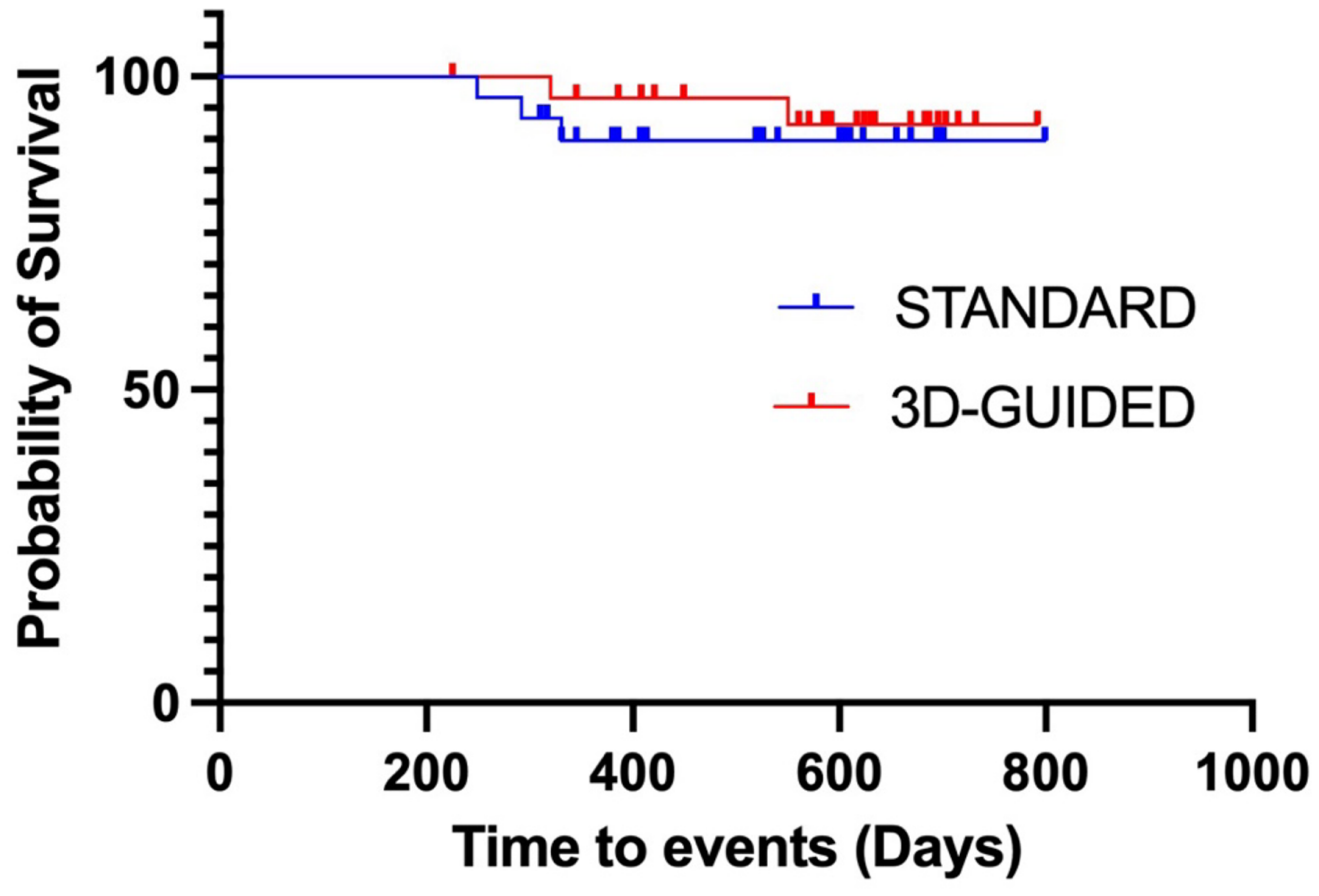
Kaplan–meier curve of atrial tachyarrhythmia (ATa)-free survival during follow-up; the freedom from ATAs was 89.7% and 92.3% for the FLUO and 3D-EAM groups, respectively.

Sixty patients out of AAD fulfilled the follow-up protocol and were included in the survival analyses. With a median follow-up of 579.0 (402–632) days, the ATA-free rates were 89.7% and 92.3% in the FLUO and 3D-EAM groups, respectively (*P* > 0.05; Figure 1).

**Figure 2:**
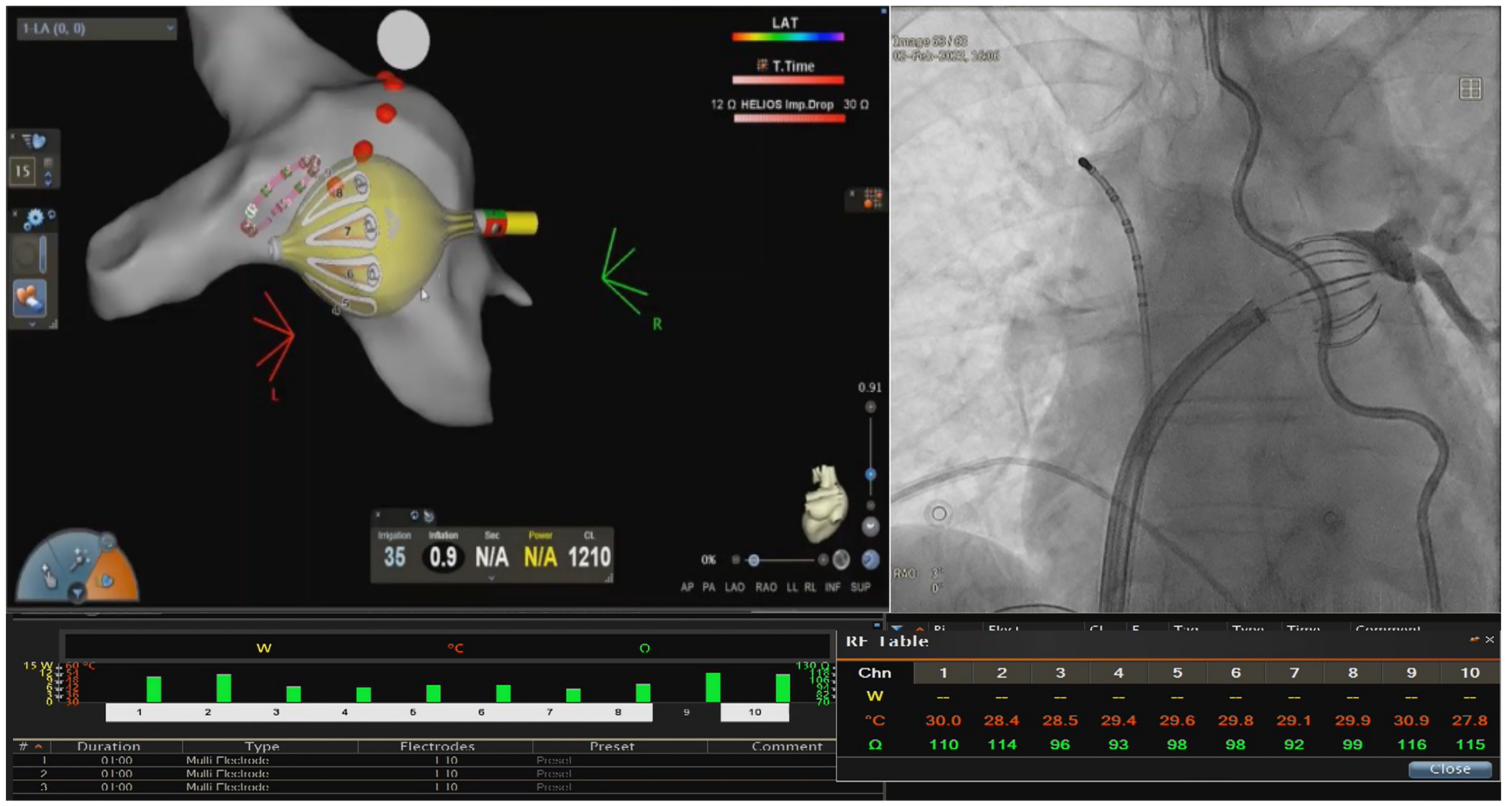
Example of RFB positioning. Example of optimal balloon positioning in the left inferior pulmonary vein: inflation index >0.8; baseline impedance across 10 electrodes ranging between 80 and 120 Ω; and baseline temperature <31°C in all electrodes. Electrodes #6, #7, and #8 are selected as posterior electrodes.

### Mandated remap

Thirty patients (15 from each group) out of AAD underwent the mandated remap procedure after a median of 177 (IQR, 145–192) days. Durable PVI on a per PV basis was present in 54/60 (90%) vs. 57/60 (94%) PVs in the FLUO and 3D-EAM groups, respectively (*P* = 0.9). The antral level of isolation was similar in both groups. PVI on a per-patient basis was present in 24 out of 30 patients (80.0%), without any significant difference between groups (12 from each group). The distribution of late PV reconnection sites was five right superior PVs, two right inferior PVs, one left superior PV, and one left inferior PV. All reconnected PVs were successfully reisolated.

### Temperature and impedance profile analysis

When comparing the FLUO and 3D-EAM groups, there were no differences in baseline impedance, impedance drop, baseline temperature, or temperature rise for either the posterior or anterior electrodes. Detailed metrics are shown in Appendix A.

## Discussion

The study analysis highlights that 3D mapping-guided positioning of the RFB optimises the safety profile with comparable efficiency and thermal characteristics for PVI. This study can be summarised as follows: (1) 3D mapping-guided RFB positioning for PVI resulted in comparable freedom from recurrent ATAs when compared with standard fluoroscopy-guided balloon positioning; (2) efficacy parameters, including lesion metrics, single-shot isolation rates, and TTI, were comparable between the FLUO and 3D-EAM groups, demonstrating no compromise in procedural effectiveness; (3) the 3D mapping-guided balloon positioning strategy was characterized by shorter fluoroscopy exposure; and (4) the high rate of durable PVIs (≥90%) were comparable at 145 days in both groups.

### Long-term outcomes and follow-up

Long-term follow-up data revealed comparable rates of ATA-free survival between both groups. Indeed, after a follow-up of 19.0 (13.2–20.8) months, the overall freedom from ATA recurrence without AAD was identical in both groups (89.7% for FLUO and 92.3% for 3D-EAM, *P* > 0.05), with no difference in recurrence type.

These results align with recent studies investigating various ablation techniques. Previous studies reported freedom from ATA rates ranging from 78.2% to 87% at 12 months, including the RADIANCE (12), FIRE AND ICE (13), Close to CURE (14), and CIRCA-DOSE trials (15); newer technologies, like pulsed-field ablation (PFA), have achieved a freedom from ATA rate of 84.5% (16).

These findings suggest that positioning the RFB with 3D-mapping guidance during ablation does not compromise PVI durability, as evidenced by sustained rhythm control over extended follow-up periods.

### Procedural outcomes

PVI represents a fundamental approach to AF ablation therapy. As the demand for AF treatment increases, there is a notable shift towards more efficient techniques, particularly those employing single-shot technologies. Cryoballoon ablation (CBA) has emerged as the conventional method (2), yet a novel single-shot catheter, the RFB, has been recently introduced. This advanced balloon technology distinguishes itself from prior methods by utilizing RF energy instead of cryoablation and integrating it with a 3D EAM system (14, 15, 17, 18). Compared with the established single-shot cryoballoon approach, RFB demonstrates comparable safety, efficacy, and efficiency metrics but with reduced dwell and thermal delivery times (4, 17, 19).

In addition, RFB integrates a 3D electroanatomical mapping system, allowing different metrics to measure correct balloon position while relying less on fluoroscopy. This could lead to more consistent proper balloon positioning and reduce patient irradiation (11).

The results of this study indicate that positioning the balloon during PVI procedures using specific temperature and impedance thresholds, thanks to the 3D-mapping system, significantly reduced dye consumption to zero and fluoroscopy exposure from 10.5 to 7.0 min (*P* < 0.05), without compromising procedural efficacy. Avoiding dye injection could be considered an added value in diabetic, kidney failure, and elderly patients. On the other hand, fluoroscopy time in the FLUO group aligns with the literature-reported value of 8–16 min, while fluoroscopy time in the 3D-EAM group was 30% shorter, 5–14 min. Importantly, reducing fluoroscopy time is a significant benefit for patient health as it minimises exposure to ionising radiation, thereby lowering the risk of radiation-related complications and long-term adverse effects (20). Single-shot isolation presented comparably high rates across all PVs, with a mean of 90.7% for the FLUO group and 94.7% for the 3D-EAM group, and a median TTI of 9.0 (7.5–10.5) and 9.5 s (7.7–10.5), respectively. These metrics were also comparable to values reported by other trials (21, 22).

### Comparison with previous remapping studies

Trials requiring invasive remapping procedures, regardless of arrhythmia recurrence, are challenging to conduct due to the lack of willingness of asymptomatic patients to undergo a second procedure. Therefore, most data on chronic PVI durability derive from studies where a repeat procedure was performed only if symptoms recurred, which does not provide accurate feedback on overall PVI durability (23–25). There are few studies in which an intentional, protocol-mandated, invasive repeat procedure was performed to assess the durability of the PVI. The published rates of durable isolation on a per-vein and per-patient basis vary widely. For CB technology, the SUPIR study, involving 19 patients, reported a durable isolation of 79% per patient and 91% per PV after six months (26). For RF technologies, the results varied from 62.5% per patient and 85% per PV (the EFFICAS II study) (27), 37.5% per patient and 74% per PV (The PRESSURE study) (28), 31% per patient (the LOCALIZE study) (9), 72.5% per patient and 90% per PV (the HPSD remap study) (29), and 78% per patient and 93% per durable PVI (the PRAISE study) (30). The number of patients varied from 20 to 50 and the remap was planned two to three months after the index procedure.

For PFA, the combined IMPULSE, PEFCAT I, and PEFCAT II studies, involving 110 patients, presented a durable isolation of 65% per patient and 85% per PV at two-three months (16).

In the current study, despite the complexity of the design arising from the mandatory repeat procedure, 30 patients underwent initial PVI either with fluoroscopy-guided or 3D mapping-guided balloon positioning and completed the six-month remapping procedure. After a median of six months, these 30 patients (15 in each group) underwent a high-density left atrial remap, showing durable PVI in 80.0% of patients (80% fluoroscopy-guided and 80% 3D mapping-guided) and 92% of PVs (90% and 94%, respectively). Our results are similar to those of the SUPIR study, where the index procedure was performed with a similarly advanced ablation tool and protocol (26). Of note, we performed the repeat procedure at six months rather than the three months of the SUPIR study. Moreover, we used high-density mapping for the repeat procedure after initial RF ablation, which might enhance the detection of localised reconnection sites. In addition, both groups demonstrated similar levels of isolation, suggesting that fluoroscopy-guided complete dye occlusion of the PVs is comparable to impedance/temperature feedback-based occlusion (31). The architecture of the RFB (compliant, 10 large electrodes, irrigation on each electrode) and the integration of real-time impedance/temperature feedback within the RFB technology may ensure consistent lesion formation and durable lesions.Finally, the location and distribution of the reconnections observed in the current study align with those of previous studies, with right PVs being the most frequent site of reconnection with RF ablation (32, 33).

### Lesion metrics and thermal characteristics

Previous studies recommended achieving post-ablation impedance drops exceeding 12 Ω and temperature increases greater than 6°C (6, 12, 34). Del Monte et al., in a recent publication, suggested that achieving an impedance drop exceeding 19.2 Ω and a temperature rise exceeding 11.1°C may serve as potential predictors of acute, persistent single-shot isolation (6). It is interesting to note that ablation and post-ablation parameters were similar across electrodes between the two groups, indicating that the level of occlusion with both methods is sufficiently comparable to lead to an efficient impedance drop and temperature rise. This similarity is also likely attributable to the operator's high level of experience with RFB procedures. These findings suggest that while experienced clinicians achieve similar outcomes with both methods, 3D-mapping guidance might constitute a valuable tool for less experienced operators, reducing the reliance on fluoroscopy, smoothing out the learning curve, and improving procedural safety.

### Safety profiles and complications

This prospective study encountered no major complications in any group, including pericardial effusion, stroke, TIA, atrial-esophageal fistulas, or PV stenosis.

Regarding minor complications, a transient phrenic nerve injury occurred in one patient from the 3D-EAM group, which resolved during follow-up visits without requiring additional treatment.

### Limitations

The main limitation of the study is its single-centre design and these results are probably mediated by the fact that our study was single centered and conducted by highly experienced operators in single-shot PVI. Notably, the procedures were evenly performed among all operators. Finally, no PV stenosis were evaluated during follow-up, however, no patients reported symptoms typically associated with PV stenosis. Future studies with larger cohorts will be essential to enhance statistical power and assess the generalizability of our findings more comprehensively.

### Clinical implications and future directions

The findings of our study underscore the significant impact of precise balloon positioning on the durability of PVI following RFB ablation. The comparison between standard fluoroscopy and 3D mapping-guided positioning reveals that the latter, with its integration of temperature and impedance measurements, decreases patient irradiation while maintaining key efficacy parameters and safety.

Additionally, regardless of the positioning technique, the durable PVIs obtained in both groups emphasise the importance of post-ablation parameters as reliable indices for long-term isolation.

## Conclusions

This study demonstrates that PVI with the RFB is durable whether it is guided by fluoroscopy alone or 3D mapping. The latter avoids dye consumption and reduces fluoroscopic times.

## Data Availability

The raw data supporting the conclusions of this article will be made available by the authors, without undue reservation.

## Appendix

**Table A1:** Comparison of lesion metric values between the FLUO and 3D-EAM groups after RF delivery.

Groups		FLUO	3D-EAM
ANT	Baseline Impedance, Ω (range)	104.03 (99.49–108.03)	104.03 (100.04–107.65)
	Delta Impedance, Ω (range)	21.41 (18.48–25.43)	20.86 (19.46–23.01)
	Baseline Temperature, °C (range)	27.88 (27.19–28.20)	27.60 (27.00–27.95)
	Delta Temperature, °C (range)	13.37 (10.69–16.05)	13.98 (12.30–14.60)
	Time to Max. Impedance, s (range)	20.84 (16.70–25.93)	21.79 (17.56–26.80)
	Time to Max. Temperature, s (range)	30.45 (26.48–36.83)	34.45 (31.40–40.24)
PST	Baseline Impedance, Ω (range)	108.09 (104.82–111.51)	110.25 (99.55–117.11)
	Delta Impedance, Ω (range)	23.06 (19.42–30.62)	28.33 (19.26–32.21)
	Baseline Temperature, °C (range)	27.43 (26.44–28.09)	26.88 (26.13–27.38)
	Delta Temperature, °C (range)	11.00 (10.39–14.33)	11.58 (9.41–14.91)
	Time to Max. Impedance, s (range)	12.51 (11.42–16.23)	16.09 (13.26–17.59)
	Time to Max. Temperature, s (range)	14.51 (13.02–16.66)	14.40 (13.93–17.15)

ANT, anterior electrode; POST, posterior electrode.

Comparison of impedance drop and temperature rise at each electrode from baseline to total RF delivery between groups. No significant differences were found.
